# The Protein Phosphatase GhAP2C1 Interacts Together with GhMPK4 to Synergistically Regulate the Immune Response to *Fusarium oxysporum* in Cotton

**DOI:** 10.3390/ijms23042014

**Published:** 2022-02-11

**Authors:** Dezheng Guo, Cuihong Hao, Junbin Hou, Guangdong Zhao, Wenlu Shan, Huijuan Guo, Chen Wang, Xingqi Guo

**Affiliations:** State Key Laboratory of Crop Biology, College of Life Sciences, Shandong Agricultural University, Taian 271018, China; gzgdz666@163.com (D.G.); cuihonghao12@126.com (C.H.); houjunbin123@163.com (J.H.); guangdong188@163.com (G.Z.); shanwenlu33@163.com (W.S.); hjguo0906@163.com (H.G.)

**Keywords:** MAPK cascade, plant immune responses, protein phosphatase, cotton, *Fusarium oxysporum*, plant-microbe interactions

## Abstract

The plant mitogen-activated protein kinase (MAPK) cascade plays an important role in mediating responses to biotic and abiotic stresses and is the main pathway through which extracellular stimuli are transduced intracellularly as signals. Our previous research showed that the GhMKK6-GhMPK4 cascade signaling pathway plays an important role in cotton immunity. To further analyze the role and regulatory mechanism of the GhMKK6-GhMPK4 cascade signaling pathway in cotton resistance to *Fusarium* wilt, we functionally analyzed GhMPK4. Our results show that silencing GhMPK4 reduces cotton tolerance to *Fusarium* wilt and reduces the expression of several resistance genes. Further experiments revealed that GhMPK4 is similar to GhMKK6, both of whose overexpression cause unfavorable cotton immune response characteristics. By using a yeast two-hybrid screening library and performing a bioinformatics analysis, we screened and identified a negative regulator of the MAPK kinase-protein phosphatase AP2C1. Through the functional analysis of AP2C1, it was found that, after being silenced, GhAP2C1 increased resistance to *Fusarium* wilt, but GhAP2C1 overexpression caused sensitivity to *Fusarium* wilt. These findings show that GhAP2C1 interacts together with GhMPK4 to regulate the immune response of cotton to *Fusarium oxysporum*, which provides important data for functionally analyzing and studying the feedback regulatory mechanism of the MAPK cascade and helps to clarify the regulatory mechanism through which the MAPK cascade acts in response to pathogens.

## 1. Introduction

During their growth and development, plants can experience damage from bacteria, fungi, and viruses [1,2,3,4]. Throughout the long-term evolutionary process, higher plants have developed a variety of immune responses to resist the infection of pathogenic bacteria to survive, including constitutive preformed and induced defense responses, providing several layers of protection against pathogen invasion [1,2,3]. The innate immune response is a type of induced defense composed of pathogenic-associated molecular pattern (PAMP)-triggered immunity (PTI) and effector-triggered immunity (ETI) [1,5,6,7,8]. PTI is the first layer of innate immunity, in which pattern recognition receptors (PRRs) sense conserved molecules or structures of pathogens and activate downstream defense responses [6,9,10]. ETI is the second layer of innate immunity. Pathogens can transfer effector proteins into host cells, which then interfere with the defense response. Hosts directly or indirectly recognize these highly variable effectors and in turn induce a disease resistance response [3,11,12]. Unlike studies involving the initially proposed classic zigzag model [1] of plant immunity, recent groundbreaking studies have shown that PTI and ETI can interact together as part of the plant immune system [13,14]. The mitogen-activated protein kinase (MAPK) cascade signaling pathway plays a key role in plant-microbe interactions, with PTI using MAPKs and NADPH signaling to ensure proper ETI-integrated resistance function [13,14].

Signal transduction networks constitute an important component of plant immune systems [9,15]. The mitogen-activated protein kinase (MAPK) cascade signaling pathway plays a central role in the signaling pathway that converts extracellular stimuli into intracellular responses in eukaryotes [16]. When infected by pathogenic bacteria, plants use PRRs to quickly recognize conserved PAMPs of pathogens and activate receptor-like cytoplasmic kinases (RLCKs) by binding to coreceptors and via phosphorylation, thereby activating MAPK cascade signaling pathways and calcium-dependent protein kinase (CDPK) pathways and causing reactive oxygen species (ROS) bursts [17,18]. The ETI response can also activate the MAPK signaling pathway and cause ROS bursts [19]. Activation of the MAPK cascade, the central node of the intersection of PTI and ETI, regulates a variety of immune responses, such as the production of ROS and the stress/defense hormones salicylic acid (SA), jasmonic acid (JA) and ethylene (ET) [20,21,22].

The MAPK cascade signaling pathway is highly conserved in eukaryotes [21]. A typical MAPK cascade consists of three sequentially activated protein kinases (MAPKKK, MAPKK, MAPK) that transduce signals in the form of a phosphorylation cascade, and this cascade induces physiological and biochemical responses [23,24]. In plants, the MAPK cascade pathway is related to various physiological, developmental and hormone responses [20,21,24,25]. Studies using biochemical and molecular biology methods have shown that MAPK cascades can be activated by various stimuli, such as pathogen infection and mechanical damage [26,27]. Various MAPK signal transduction pathways that respond to different stimuli have been identified [23,24]. For example, the MEKK1-MKK1/2-MPK4 cascade is involved in responses to cold stress, salt stress and pathogen infection [28,29]. AtMPK4 is a widely studied MAPK gene, and in *Arabidopsis thaliana*, MAPKKK1, MKK1/MKK2, and MPK4 play a role in the MAPK cascade and regulate innate immunity [30].

Among *Arabidopsis thaliana* protein phosphatase type 2Cs (PP2C) family members, AP2C proteins have a kinase-interacting motif (KIM) that is highly similar to the KIM motif found in mammalian MAPK phosphatase [31,32]. The specificity of the interaction between AP2Cs and MAPKs has been confirmed [33,34]. AP2C1 regulates MAPK activity induced by trauma and PAMPs and its overexpression can impair stress-induced ET production and plant resistance to the necrotic fungus *Botrytis cinerea* [34,35], PAMP sensing strongly induces the expression of AP2C1 and its nearest homologous gene AP2C2 in *Arabidopsis thaliana* [36,37]; AP2C1 is structurally and functionally different from dual-specificity phosphatase [37,38]. PP2C-type phosphate enzymes such as *Arabidopsis thaliana* AP2C1, AP2C2, AP2C3 and AP2C4 also dephosphorylate MAPKs [39,40]. In addition, the alfalfa AP2C1 homologous gene MP2C inactivates the MPK6 homologous gene SIMK through dephosphorylating the pTEpY motif [41].

Cotton is one of the most economically important crop species worldwide, but its yield is limited by a variety of biotic and abiotic stresses [42,43]. *Fusarium* wilt, caused by invasion of *Fusarium oxysporum*, is one of the most severe diseases of cotton [44,45]. As such, resistance to *Fusarium oxysporum* has important practical significance. Our previous research showed that the GhMKK6-GhMPK4 cascade signaling plays an important role in cotton immunity [46]. However, we also found that GhMKK6 overactivation can cause allergic-like cell death, resulting in a disease-like phenotype [47].

In order to further analyze the role and regulatory mechanism of the GhMKK6-GhMPK4 cascade signaling pathway in cotton resistance to *Fusarium* wilt, in this study, we analyzed the function of GhMPK4 and found that GhMPK4 is similar to GhMKK6. Moreover, when they are overexpressed, both cause unfavorable cotton immune response characteristics. We speculate that there may be a negative regulatory factor in cotton that can finely tune the negative effects of GhMPK4. Using a yeast two-hybrid screening library, we screened a protein with the GeneID gene number: 107893381. After using a variety of bioinformatics methods, we found that this protein has a typical KIM motif; thus, the protein is a AP2C protein. We further used yeast two-hybrid, glutathione S-transferase (GST) pulldown, and luciferase complementation imaging (LCI) assays, which verified that GhAP2C1 and GhMPK4 interact. Through functional analysis of AP2C1, it was found that GhAP2C1 silencing increased resistance to *Fusarium* wilt but that GhAP2C1 overexpression caused sensitivity to *Fusarium* wilt. Our results compensate for the lack of knowledge on the pathogenic process and provide an important scientific basis for cotton *Fusarium* wilt prevention strategies.

## 2. Results

### 2.1. Enhanced Sensitivity of GhMPK4-Silenced Cotton to Fusarium oxysporum Increased

**Figure 1:**
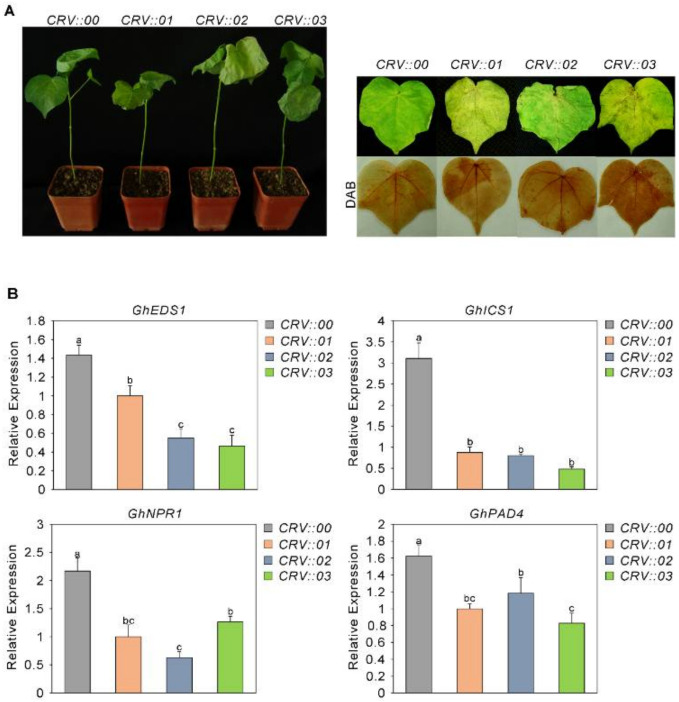
Enhanced sensitivity of GhMPK4-silenced cotton to *Fusarium oxysporum* increased. (**A**) Representative phenotypes of GhMPK4-silenced cotton after infection with *Fusarium oxysporum* for 7 days. DAB, diaminobenzidine. (**B**) Expression level of salicylic acid (SA)-mediated defense pathway genes and pathogenesis-related genes in GhMPK4-silenced cotton plants after infection with *Fusarium oxysporum* for 7 days. The error bars indicate the mean ± SEs of three independent experiments (*n* = 9). The letters above the columns represent significant differences (*p* < 0.05) based on Tukey’s HSD test.

Our previous research showed that the MKK6-MPK4 cascade plays an important role in the immune response of cotton to *Fusarium oxysporum* [46], and we found that GhMKK6 overactivation caused an excessive hypersensitive response (HR) [47]. To further analyze the immune response of cotton challenged with *Fusarium oxysporum*, we studied the function of GhMPK4 downstream of GhMKK6. We used virus-induced gene silencing (VIGS) technology to silence cotton GhMPK4. After silencing was performed, cells were inoculated with an *Fusarium oxysporum* spore suspension (10^6^ conidia/mL). Seven days later, the incidence of *Fusarium* wilt was measured, and it was found that GhMPK4-silenced cotton plants were sensitive to *Fusarium oxysporum* (Figure 1A). As shown in Figure 1A, compared to CRV::00, the infected leaves withered more severely.

We then analyzed the expression patterns of several genes involved in the SA-mediated defense pathway. As shown in Figure 1B, compared with those in CRV::00 leaves, the SA-mediated expression levels of genes (EDS1, ICS1, NPR1, PAD4) in the infected leaves were significantly reduced. Taken together, these results suggest that GhMPK4 may be related to the SA-mediated defense pathway and thus may play an important role in cotton defense against fungi.

### 2.2. The Sensitivity of Plants to Fusarium oxysporum Increased after the Overexpression of GhMPK4

**Figure 2:**
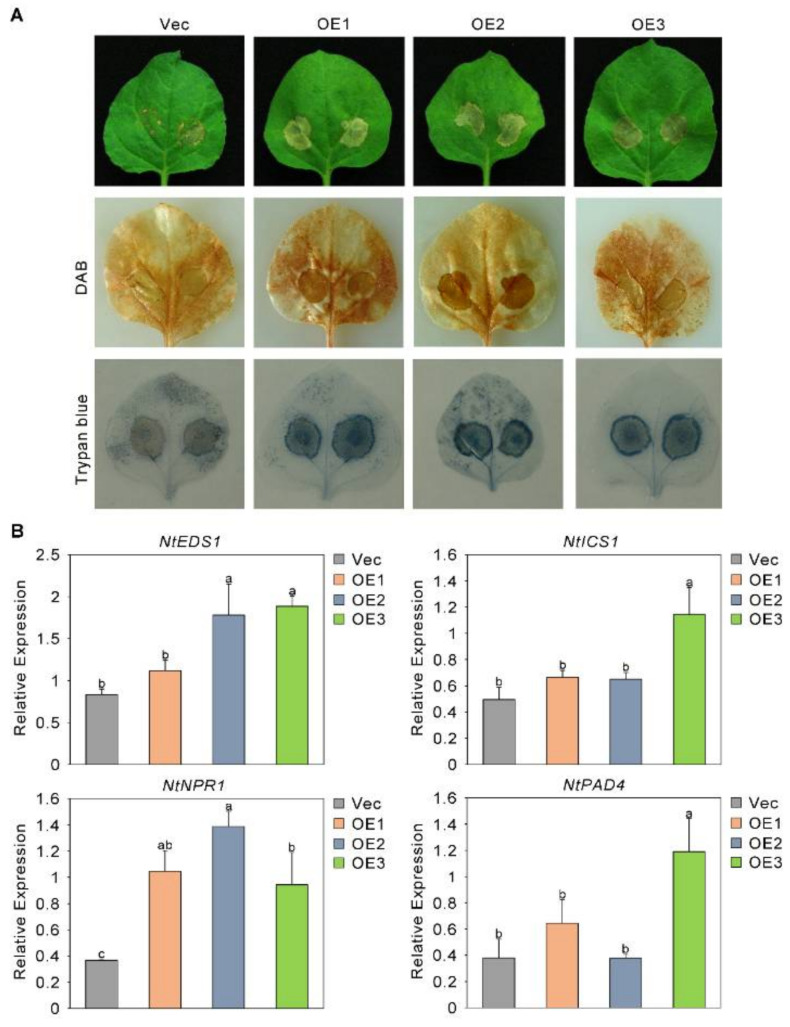
The sensitivity of plants to *Fusarium oxysporum* increased after the overexpression of GhMPK4. (**A**) Representative phenotypes of GhMPK4-overexpressing plants after infection with *Fusarium oxysporum* for 7 days. Vec, a empty vector control line; OE, GhMPK4-overexpressing transgenic plants; DAB, diaminobenzidine. (**B**) Expression level of salicylic acid (SA)-mediated defense pathway genes and pathogenesis-related genes in GhMPK4-overexpressing plants after infection with *Fusarium oxysporum* for 7 days. The error bars indicate the mean ± SEs of three independent experiments (*n* = 9). The letters above the columns represent significant differences (*p* < 0.05) based on Tukey’s HSD test.

To further analyze the role of GhMPK4 in the immune response to *Fusarium oxysporum*, we transferred GhMPK4 into *Nicotiana benthamiana* with different expression levels—low, medium, and high (referred to as OE1, OE2, and OE3, respectively). The biological function of the GhMPK4 gene was further studied. The results showed that at 7 days after infection, the degree of leaf wilting of the overexpression (OE) plants was more severe than that of the control plants. Trypan blue and 3,3′-diaminobenzidine (DAB) histochemical staining revealed that the leaves of the OE plants accumulated more blue and brown staining than the leaves of the Vec plants did (Figure 2A). We then analyzed the expression patterns of several genes involved in the SA-mediated defense pathway. As shown in Figure 2B, the expression of the target gene increased in the OE leaves. Taken together, these results imply that overexpression of GhMKK6-GhMPK4 causes an excessive HR.

### 2.3. Characterization of GhAP2C1

**Figure 3:**
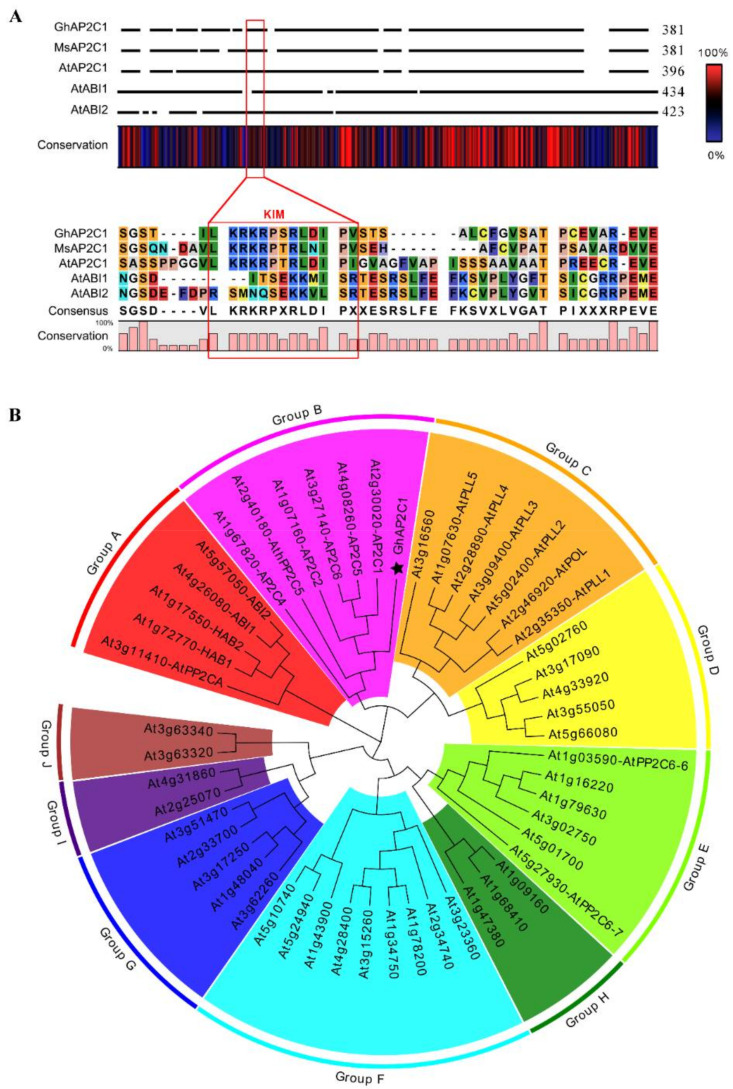
Characterization of GhAP2C1. (**A**) Amino acid sequence alignment and motif analysis of GhAP2C1. KIM means kinase interacting motif that contains the “LKRKRPXRLDIPX” amino acid sequence. (**B**) GhAP2C1 Dendrogram analysis of all PP2Cs families in *Arabidopsis thaliana*. GhAP2C1 is highlighted using ☆. Group A-J indicate PP2Cs groups.

During their evolution, plants have developed a strict feedback regulatory mechanism. We suspect that there is a negative regulatory mechanism in plants to alleviate GhMKK6-GhMPK4 overactivation that causes an excessive HR. We used GhMPK4 as bait to generate a yeast two-hybrid screening library. Through yeast two-hybrid screening, we noticed a protein with gene number of GeneID: 107893381 in cotton. Phylogenetic and comparative multiple sequence analyses showed that it is extremely highly homologous to AtAP2C1 of the D family of *Arabidopsis thaliana* PP2Cs (Figure 3). Therefore, we named it GhAP2C1. Further motif analysis revealed that GhAP2C1, along with MP2C, the earliest protein phosphatase found in plants, and AtAP2C1 all contain a typical KIM structural domain (Figure 3A).

### 2.4. Verification of GhAP2C1 and GhMPK4 Interactions

**Figure 4:**
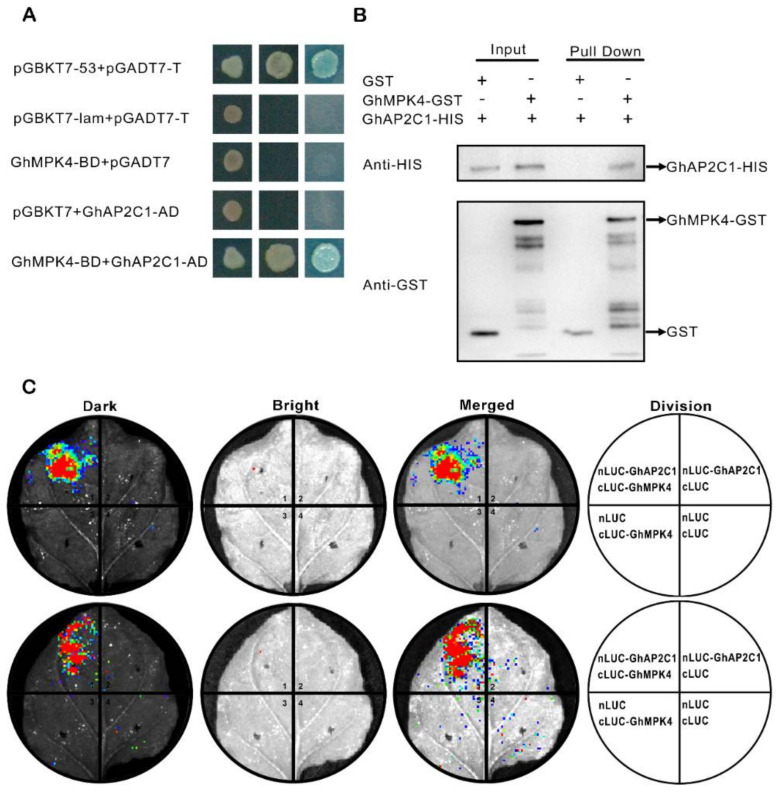
Verification of GhAP2C1 and GhMPK4 interactions. (**A**) GhMPK4 specifically interacts with GhAP2C1 according to yeast two-hybrid system assay results. The indicated BD and AD fusion constructs were co-transformed into the Y2H Gold yeast strain and grown on the SD media DDO and QDO/X. SD, synthetically defined medium; SD medium DDO, SD medium without Leu and Trp; SD medium QDO/X, SD medium with X-a-gal and without Ade, His, Leu, Trp. (**B**) Luciferase (LUC) complementation imaging (LCI) assay shows that GhMPK4 could interact with GhAP2C1 in Nicotiana benthamiana. Signals were only detected with the co-transformation of GhMPK4 andGhAP2C1. Nicotiana benthamiana leaves were co-infiltrated by *Agrobacterium tumefaciens* strain GV3101 containing the indicated construct pairs. The signals were detected 48 h after infiltration. (**C**) GST pull-down assay shows that GhMPK4 could interact with GhAP2C1 in vitro. Cell lysates of *Escherichia coli* expressing GhAP2C1-HIS were co-incubated with cell lysates of *E. coli* expressing GST or GhMPK4-GST and glutathione-sepharose beads. Western blot reacted with anti-HIS and anti-GST antibodies.

To verify the findings of our yeast two-hybrid screening library, we first verified the interaction between GhAP2C1 and GhMPK4 through a yeast two-hybrid experiment. As shown in the Figure 4A, the positive control also had GhMPK4 and GhAP2C1 on a plate containing synthetic dropout (SD) media. In addition, the transformed clones grew well on double dropout supplement (DDO) and quadruple dropout supplement (QDO) media, indicating that GhMPK4 and GhAP2C1 interact. To further verify the interaction between GhAP2C1 and GhMPK4, we conducted GST pulldown (Figure 4B) and LCI (Figure 4C) experiments. The results show that GhAP2C1 interacts together with GhMPK4.

### 2.5. Expression Characteristics of GhAP2C1 and GhMPK4

**Figure 5:**
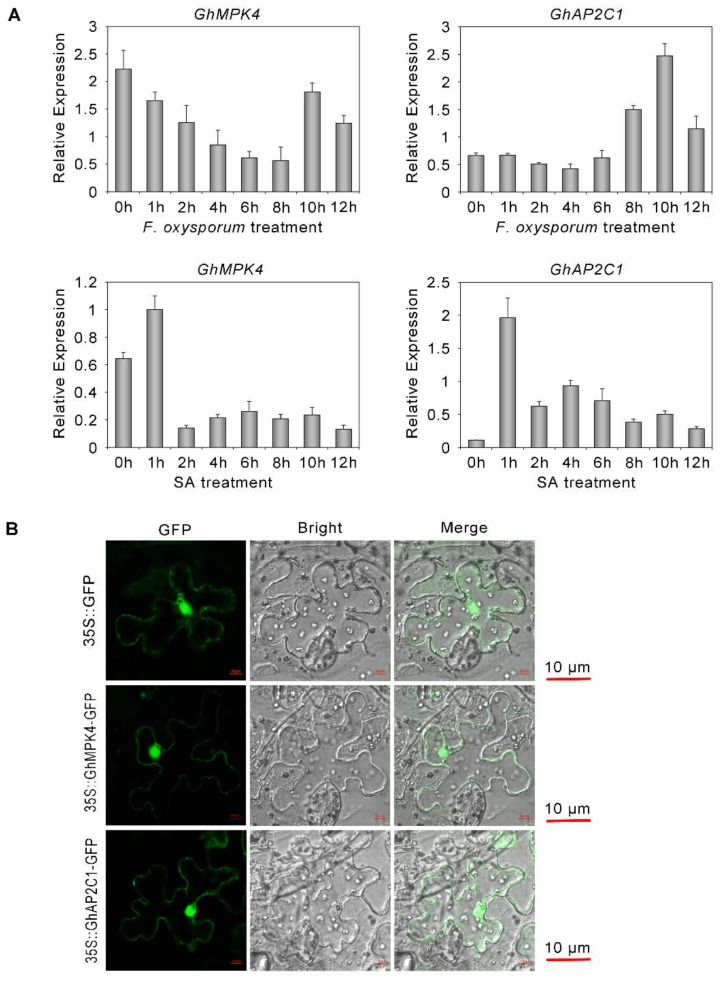
Expression characteristics of GhAP2C1 and GhMPK4. (**A**) qRT-PCR analysis of GhMPK4 and GhAP2C1 expression under salicylic acid (SA) or *Fusarium oxysporum* treatment. (**B**) Subcellular localization of GhMPK4 and GhAP2C1 in *Nicotiana benthamiana* leaves. Green fluorescence was observed with an LSM 880 META confocal microscope (Carl Zeiss). GFP, green fluorescent protein.

To determine whether GhAP2C1 is involved in the immune response of cotton to *Fusarium oxysporum* and to further explore the relationship between GhAP2C1 and GhMPK4, we measured the transcriptional changes of GhAP2C1 and GhMPK4 after inoculation and SA treatment. As shown in the Figure 5A, like GhMPK4, GhAP2C1 responds to *Fusarium oxysporum* infection. SA treatment resulted in a significant decrease in the expression of GhMPK4, and the expression of GhAP2C1 increased. In addition, we further experimented and found that GhAP2C1 and GhMPK4 have the same subcellular localization (Figure 5B). These results show that GhAP2C1 may also play an important role in the signal transduction pathway of plant disease resistance.

### 2.6. The Sensitivity of GhAP2C1-Silenced Cotton to Fusarium oxysporum Increased

**Figure 6:**
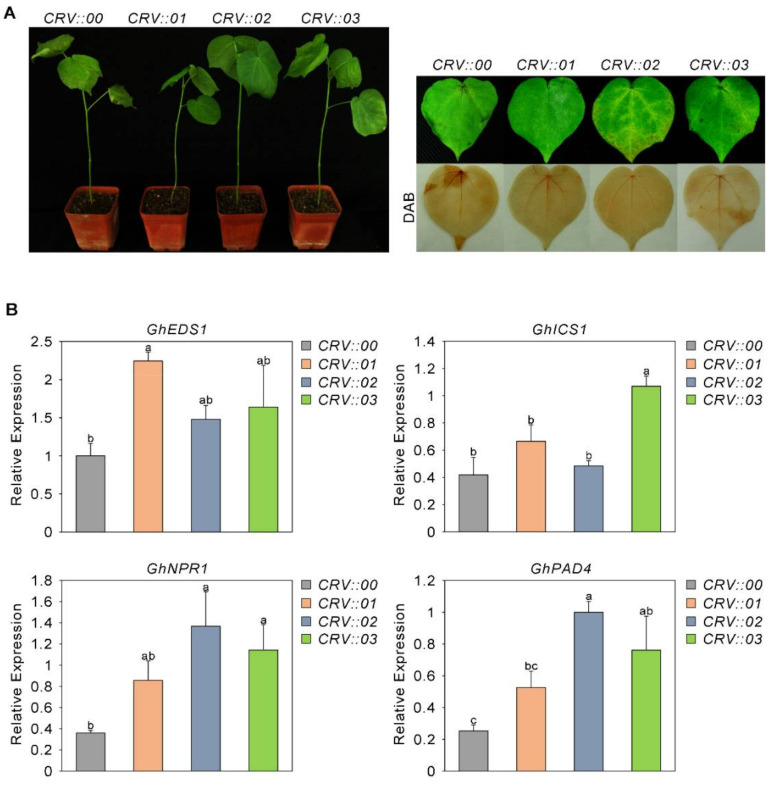
The sensitivity of GhAP2C1-silenced cotton to *Fusarium* oxysporum increased. (**A**) Representative phenotypes of GhAP2C1-silenced cotton after infection with *Fusarium oxysporum* for 7 days. DAB, diaminobenzidine. (**B**) Expression level of salicylic acid (SA)-mediated defense pathway genes and pathogenesis-related genes in GhAP2C1-silenced cotton plants after infection with *Fusarium oxysporum* for 7 days. The error bars indicate the mean ± SEs of three independent experiments (*n* = 9). The letters above the columns represent significant differences (*p* < 0.05) based on Tukey’s HSD test. Same letter indicates no significant difference; different letters indicate significant differences. If a non-significant difference was found between two groups, overlapping letters are used to indicate the statistical significance.

To analyze the role of GhAP2C1 in the cotton immune response to *Fusarium oxysporum*, like we did for GhMPK4, we used VIGS technology to silence cotton GhAP2C1 and then infected cotton with a suspension of *Fusarium oxysporum*. After 7 days, the plants were evaluated. The occurrence of *Fusarium* wilt revealed that the GhAP2C1-silenced cotton plants had increased resistance to *Fusarium oxysporum* (Figure 6A). We then analyzed the expression patterns of several genes involved in the SA-mediated defense pathway. As shown in Figure 6B, compared with that in the leaves of CRV::00 plants, the expression level of SA-mediated genes (EDS1, ICS1, NPR1, PAD4) in the leaves of infected plants significantly increased. These results suggest that GhAP2C1 may interact with components of the SA-mediated defense pathway and may play an important role in the cotton defense against fungi.

### 2.7. The Sensitivity of Plants to Fusarium oxysporum Increased after the Overexpression of GhAP2C1

**Figure 7:**
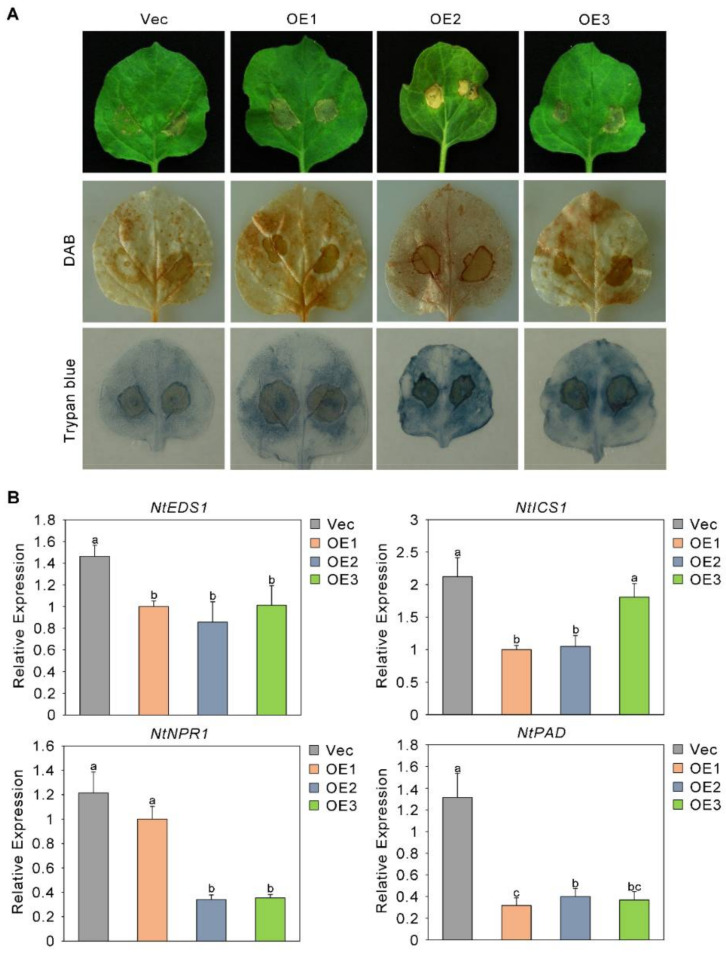
The sensitivity of plants to *Fusarium* oxysporum increased after the overexpression of GhAP2C1. (**A**) Representative phenotypes of GhAP2C1-overexpressing plants after infection with *Fusarium* oxysporum for 7 days. DAB, diaminobenzidine. (**B**) Expression level of salicylic acid (SA)-mediated defense pathway genes and pathogenesis-related genes in GhAP2C1-overexpressing plants after infection with *Fusarium* oxysporum for 7 days. The error bars indicate the mean ± SEs of three independent experiments (*n* = 9). The letters above the columns represent significant differences (*p* < 0.05) based on Tukey’s HSD test.

Like we did for GhMPK4, to further test the function of GhAP2C1, we further tested the biological function of GhAP2C1 in cotton against *Fusarium oxysporum* by overexpressing GhAP2C1 in *Nicotiana benthamiana*. At 7 days after infection, *Fusarium oxysporum* had a significant effect on the plants. Compared with the Vec plants, the OE plants exhibited more severe leaf wilting, and their symptoms and leaf yellowing were more severe. In addition, trypan blue and DAB histochemical staining showed that leaves of OE plants accumulated more blue and brown staining than did the leaves of the Vec plants (Figure 7A). We then analyzed the expression patterns of several genes involved in the SA-mediated defense pathway. As shown in Figure 7B, the expression of the target gene decreased in the leaves of the OE plants. Taken together, these results show that overexpression of GhAP2C1 is not conducive to the immune response of plants to *Fusarium oxysporum*.

## 3. Discussion

The MAPK pathway is one of the most important signal transduction pathways in plants [23,48]. By converting extracellular stimuli into intracellular responses, the MAPK cascade plays a central role in signaling in eukaryotes. [49]. Numerous studies have revealed the important role of the MAPK cascade in plant immunity. Among them, the MEKK1-MKK4/MKK5-MAPK3/MAPK6 pathway was the earliest identified MAPK cascade signaling pathway, and activation of this MAPK cascade can improve resistance to bacteria. [50]. In our previous research, we found that the cotton GhMKK6-GhMPK4 signaling pathway plays an important role in cotton immunity [46]. However, we also found that GhMKK6 overactivation can cause allergic-like cell death and generate a disease-like phenotype [47]. Coincidentally, in *Arabidopsis thaliana*, constitutively activated AtMKK9 can increase the sensitivity of transgenic plants to salt tolerance [51]. However, transgenic plants overexpressing AtMKK2 showed significantly increased sensitivity to the Chlorella necrotic fungi [52]. Other studies have shown that overexpression of GhMKK5 in *Nicotiana benthamiana* increases plant resistance to *Solanomonas* bacterial pathogens but increases plant susceptibility to *Phytophthora* oomycete pathogens [53]. These findings suggest that the MAPK cascade signaling pathway is very complex [50,54,55]. Further research into the MAPK cascade signaling pathway in other plants is therefore important for understanding the molecular mechanisms of plant diseases.

To further analyze the role and regulatory mechanism of the GhMKK6-GhMPK4 signaling pathway in cotton resistance to *Fusarium* wilt, we functionally analyzed GhMPK4 downstream of GhMKK6. We used VIGS technology to silence GhMPK4 in cotton, and our results show that silencing GhMPK4 reduces cotton tolerance to *Fusarium oxysporum*. After GhMPK4 was silenced, the sensitivity to *Fusarium oxysporum* increased, and the expression of several resistance genes decreased, including EDS1, PAD4 and ICS1 (Figure 2). Further research on the function of MPK4 showed that, when overexpressed, MPK4 produces a disease-like phenotype, but the expression of the related disease resistance genes is not downregulated. These findings are similar to those of our previous study, which showed that the activation of GhMKK6 can cause allergic-like cell death and produce similar lesions [46]. This implies that, downstream of MKK6, the MPK4 expression and functional characteristics are similar to those of MKK6. In *Arabidopsis thaliana*, it has also been shown that the cascade MEKK1-MKK1/MKK2-MPK4-MKS1/WRKY33 negatively regulates the immune response in plants [56].

Plants have a systematic and complex signaling network. The members of four (I, II, III, and V) of the six subfamilies of plant MAPKs are involved in defense responses [21,23,32]. With the emergence of new information, the MAPK cascade in plant defense signaling pathways has become increasingly complicated, and the sharing of individual components in different cascades may cause crosstalk between different paths [23,57]. In addition, a given stimulus may activate multiple MAPK pathways, while different stimuli can activate the same pathway. MAPK cascade in plants involves redundancy of signaling elements, antagonism between different pathways, and positive and negative regulatory mechanisms [23]. In the signaling pathway, feedback regulation is an important mechanism that controls growth and development and the stress response. In the absence of pathogens, the defense response of plants must be strictly controlled to prevent excessive autoimmune activation [58]. Recent reports have shown that SA signal dysregulation can lead to hypersensitive response (HR) to cell death and can lead to the generation of pathological mimic phenotypes [59,60]. Similar to the SA-mediated defense response, pathogen-activated respiratory bursts are also related to the control of the HR [61]. An uncontrolled HR is harmful to plants. Compared with wild-type plants, *Arabidopsis thaliana* plants with enhanced MPK4 kinase activity showed a reduced level of resistance [62]. These results further confirm the role of MPK4 in plant defense. Based on our research results, we believe that GhMPK4 is necessary for cotton disease resistance, but similar to GhMKK6, excessive activation of MPK4 may have a negative effect. The activation degree and duration of the MAPK cascade signaling pathway are strictly controlled [56]. To prevent the excessive activation of various cellular responses, specific regulatory mechanisms are required to deactivate the MAPK cascade after it has been activated [63]. Based on the feedback regulatory mechanism, we speculate that there may be a regulatory factor in cotton that can negatively regulate GhMPK4.

We screened AP2C1 through our yeast two-hybrid screening library. After using various bioinformatics methods, we found that AP2C1 has a typical KIM motif, which suggests this protein is a member of the PP2CA family. We further used yeast two-hybrid assays, GST pulldown assays, and LCI to compare the interaction between GhAP2C1 and GhMPK4, which was confirmed. PP2Cs are also important types of phosphatases in plants. Research has shown that AP2C1 is a member of the PP2C family; AP2C1 can act as a phosphatase of MAPK to dephosphorylate threonine residues in the active region of the MAPK protein, reduce the activity of MAPK, and negatively regulate the basic immune response of plants [36]. In ap2c1 mutants, MAPK activity induced by PAMPs was significantly increased, callosin accumulation increased, and plant resistance to bacteria significantly increased [36,64]. Previous studies have confirmed that MPK3, MPK4, and MPK6 interact together with the MAPK phosphatase AP2C1, and it has been proven that the protein phosphatase AP2C1 can be used as a MAPK phosphatase to control kinase activity and signal transduction [34]. Unlike MKP, AP2C1 acts more to finely tune MAPK. Therefore, the precise and specific regulation of MAPK cascade signaling pathways by MAPK phosphatase can strongly influence a variety of plant cell responses. There is evidence that AP2C1 phosphatase plays a key role in regulating the response to stress. In *Arabidopsis thaliana*, the activity of AP2C1, which may control plant responses to gray mold, is closely related to hormone levels, the defense response and MAPK activity [36]. Our functional analysis of AP2C1 showed that GhAP2C1 participates in the immune response of cotton to *Fusarium oxysporum*. Silencing GhAP2C1 significantly enhanced the resistance of cotton to *Fusarium oxysporum*, and overexpression of AP2C1 increased sensitivity to *Fusarium oxysporum*.

In our study, we verified the biological function of GhMPK4 and further enriched the study of the MKK6-MPK4 cascade signaling pathway. We also identified and isolated a protein phosphatase, AP2C1, for the first time in cotton, and demonstrated that GhAP2C1 can coordinate with GhMPK4 to regulate the immune response to *Fusarium oxysporum* in cotton, providing important information on the functional analysis and feedback regulation of the MAPK cascade response, and helping to elucidate the mechanism of MAPK cascade response to pathogens. Our study enriches the understanding of the functions of MAPKs in cotton, further resolves the lack of knowledge in the pathogenesis of *Fusarium oxysporum*, and provides an important scientific basis for *Fusarium oxysporum* prevention.

## 4. Materials and Methods

### 4.1. Plant Treatments

Cotton (*G. hirsutum* L. cv. Lumian 22) and *Nicotiana benthamiana* were incubated in an incubator on a 16 h light/8 h dark photoperiod. For *Fusarium oxysporum* infections on cotton plants, the seedlings were inoculated with *Fusarium oxysporum* conidial suspensions (10^6^ conidia/mL) by root dip. In addition, *Fusarium oxysporum* conidial suspensions (10^6^ conidia/mL) were injected into the leaves of *Nicotiana benthamiana* overexpression plants using syringes. For expression pattern analyses, uniform cotton seedlings were sprayed with SA (10 mm) or were inoculated with *Fusarium oxysporum* conidial suspensions (10^6^ conidia/mL) by root immersion. Each treatment was repeated at least three times.

### 4.2. RNA Extraction and qRT-PCR

Total RNA was extracted using RNAiso Plus (TaKaRa, Dalian, China), cDNA was synthesized using the PrimeScript™ RT kit and gDNA Eraser (Vazyme, Nanjing, China), and qRT-PCR was performed using the SYBR^®^ PrimeScript™ RT-PCR kit (TaKaRa, Dalian, China). The UBI and β-actin genes from cotton and *Nicotiana benthamiana*, respectively, were used as standard controls. All of the experiments in this study were performed at least three times. Statistical significance between different measurements was assessed by Tukey’s honestly significant difference (HSD) test of SPSS 19.0 software. Same letter indicates no significant difference; different letters indicate significant differences. If a non-significant difference was found between two groups, overlapping letters are used to indicate the statistical significance.

### 4.3. VIGS

VIGS was performed according to the methods of Gu et al. [65]. Fragments of GhMPK4 and GhAP2C1 were inserted into a pCLCrV-A vector. pCLCrV-A and pCLCrV-B recombinant vectors were transformed into *A. tumefaciens* strain EHA105. After *A. tumefaciens* was incubated overnight, the cultures were pelleted and resuspended in infiltration media. The *Agrobacterium* suspensions containing pCLCrV-A or pCLCrV-B were mixed equally and inoculated into two fully expanded cotton cotyledons after incubation for 3 h. The leaves of the inoculated cotton plants were used for assays at three weeks after inoculation. Each assay was performed using at least three independent biological replicates.

### 4.4. Subcellular Localization of GhMPK4 and GhAP2C1

The 35S::GhMPK4-GFP, 35S::GhAP2C1-GFP and 35S::GFP recombinant plasmids were transformed into *Agrobacterium tumefaciens* strain GV3101. After incubation overnight, the cultures were pelleted and resuspended in infiltration media [10 mm MgCl_2_, 10 mM MES-NaOH (pH 3.8), 200 mm acetosyringone]. Transient expression was performed using 5-week-old *Nicotiana benthamiana* leaves. Fluorescence signals were observed with an LSM 880 META confocal microscope (Carl Zeiss, Germany).

### 4.5. Yeast Two-Hybrid, LCI and Pull-Down Assays

Interactions between GhMPK4 and GhAP2C1 were detected with a Matchmaker Gold Yeast Two-Hybrid System (Clontech, Japan). For the yeast two-hybrid system, the ORF of GhMPK4 was cloned and fused to the GAL4 DNA-binding domain in a pGBKT7 bait plasmid. The ORF regions of GhAP2C1 were separately cloned into the GAL4 activation domain in a pGADT7 prey vector. The appropriate combinations of both plasmids were co-transformed into a yeast two-hybrid Gold strain for experiment. The interaction between GhMPK4 and GhAP2C1 was assayed by luciferase complementary imaging in *Nicotiana benthamiana* leaves as described previously [66]. LUC activity in co-infiltrated leaves was observed 48 h after infiltration. GhMPK4 was expressed as a GST fusion protein in *E. coli* BL21. GhAP2C1 linked HIS-tag and was expressed in *E. coli* BL21. A 150 µL volume of bacterial protein extracts containing GST or GhMPK4-GST and 2 mL of the GhAP2C1-HIS containing protein extract were applied to 50 µL of glutathione resin. Eluates were subjected to SDS-PAGE and immunoblot analysis were performed.

### 4.6. Primers

The primers used in this study are listed in Table 1. All of the primers were synthesized by Biosune Biotechnology (Shanghai) Co., Ltd. (Shanghai, China).

## Figures and Tables

**Table 1:** Primers used in this study.

Primer	Primer Sequence (5′→3′)	Annotation
K4-QC-5	ATGAAAAAGGAAATGGGGAGTAC	The full-length cDNA primers
K4-QC-3	CTTAATGGACTGGATCTGGATTG	
AP2C1-QC-5	ATGTCGTGTTCGGTCGCTGTATG	
AP2C1-QC-3	AATATAGCGGCCCAGTTGAATC	
GhEDS1-5	GCAGCAACAGCTCCTCTACCTCAA	Primers used in qRT-PCR
GhEDS1-3	GGCAGACCAAGACGCTACAGATACA	
GhICS1-5	ATGGATGAATGGGTGCGAAGG	
GhICS1-3	AAGAATGCCAGAGGTAAGAGGAGGA	
GhPAD4-5	GGATGGAAGAATGGAAAGAAATGAA	
GhPAD4-3	GAACTAGGAAAGCAGACTAAGGAACCA	
GhNPR1-5	GCGAATCGTTGCTTTCTTCTTCA	
GhNPR1-3	CACGTGGTGCTGTTGTTGTTACTG	
NbEDS1-5	TCTGGATAGGCTGAAAGCAC	
NbEDS1-3	CCATACAAGCAAAGCAGTTCC	
NbICS1-5	CAATTCCGCCATCTCTCACT	
NbICS1-3	TGAGCATGAAGCCACTCAAG	
NbPAD4-5	GGACTCACACTCCAGCGTTT	
NbPAD4-3	GGCAACTCATCCTCTTCCTG	
NbNPR1-5	GCAGCAGACGATGTAATGATGG	
NbNPR1-3	TCCACAAGCCTAGTGAGCCTC	
AP2C1-5	GGAAGCGTTGAACTTGTCTTTG	
AP2C1-3	GCCGAAACCCCAAAACACAA	
K4-CRV-5	ACTAGTCATCAAGGACATCATTCGACC	Primers used in VIGS
K4-CRV-3	TTAATTAAGATGAACGTAATCTTTGCCTGG	
AP2C1-CRV-5	ATCAAGGCACGTCTGGGTCTCTTTTGTCAAC	
AP2C1-CRV-3	TTAATTAACTCCACTCCTGTCTCCTCTTC	
K4-BD-5	CATATGAAAAAGGAAATGGGGAGTAC	Primers used in Yeast two-hybrid
K4-BD-3	GTCGACTTAATGGACTGGATCTGGATTG	
AP2C1-AD-5	CATATGATGTCGTGTTCGGTCGCTG	
AP2C1-AD-3	GAGCTCAATATAGCGGCCCAGTTGAATC	
AP2C1-GFP-5	TCTAGAATGTCGTGTTCGGTCGCTG	Primers used in Subcellular localization
AP2C1-GFP-3	GGTACCAATATAGCGGCCCAGTTGAATC	
K4-GFP-5	TCTAGAATGAAAAAGGAAATGGGG	
K4-GFP-3	GGATCCATGGACTGGATCTGGAT	
K4-LUC-5	GGATCCATGAAAAAGGAAATGGGG	Primers used in LCI
K4-LUC-3	GTCGACATGGACTGGATCTGGAT	
AP2C-LUC-5	GGATCCATGTCGTGTTCGGTCGCTG	
AP2C-LUC-5	GTCGACAATATAGCGGCCCAGTTGAATC	
AP2C-HIS-5	GGATCCATGTCGTGTTCGGTCGCTGTATG	Primers used in Pull-down
AP2C-HIS-3	GAGCTCCTTGTCGTCATCGTCTTTGTAGTC	
K4-GST-5	GTCGACATGAAAAAGGAAATGGGG	
K4-GST-3	TCTAGACATGGACTGGATCTGGAT	

## Data Availability

Not applicable.
